# Optical imaging to map blood-brain barrier leakage

**DOI:** 10.1038/srep03117

**Published:** 2013-11-01

**Authors:** Hayder Jaffer, Isaac M. Adjei, Vinod Labhasetwar

**Affiliations:** 1 Department of Biomedical Engineering, Lerner Research Institute, Cleveland Clinic, Cleveland, OH 44195

## Abstract

Vascular leakage in the brain is a major complication associated with brain injuries and certain pathological conditions due to disruption of the blood-brain barrier (BBB). We have developed an optical imaging method, based on excitation and emission spectra of Evans Blue dye, that is >1000-fold more sensitive than conventional ultraviolet spectrophotometry. We used a rat thromboembolic stroke model to validate the usefulness of our method for vascular leakage. Optical imaging data show that vascular leakage varies in different areas of the post-stroke brain and that administering tissue plasminogen activator causes further leakage. The new method is quantitative, simple to use, requires no tissue processing, and can map the degree of vascular leakage in different brain locations. The high sensitivity of our method could potentially provide new opportunities to study BBB leakage in different pathological conditions and to test the efficacy of various therapeutic strategies to protect the BBB.

The blood-brain barrier (BBB) is a specialized vascular system comprising endothelial cell tight junctions, basal lamina, and glial processes^1^. It separates circulating blood from cerebrospinal fluid in the central nervous system (CNS)^2^. Certain drugs (e.g., nicotine and cotinine^3^), hypertonic agents (e.g., mannitol solution^4^), and cell-penetrating peptides^5^, as well as physical factors (e.g., high-intensity focused ultrasound^6^, electrical stimulation, or high-impact pressure waves^7^,^8^) can cause openings in the BBB. Disruption of the BBB is a serious condition that occurs in many pathological conditions, such as brain tumors^9^, brain injuries^10^, CNS infections^11^, neurological diseases^12^,^13^, and epilepsy^14^. In stroke, breakdown of the BBB is caused by ischemia-reperfusion injury^15^.

Since preservation of the BBB is critical in many neurological conditions, an objective, sensitive method for evaluating BBB integrity is needed. A commonly used technique is the Evans Blue (EB) assay^16^, based on the ability of EB dye to bind to serum albumin immediately following its intravenous (IV) injection into the bloodstream. Since serum albumin does not cross the BBB under normal physiologic conditions, spectrophotometric determination of EB dye accumulation in brain tissue is carried out to analyze the extent of vascular leakage^17^. However, this method requires extensive tissue processing and is not sensitive enough to detect evidence of minor leaks or to assess the global extent of vascular leakage within the brain.

Our objective is to develop a sensitive optical imaging method based on EB dye that can test BBB integrity and quantitatively map vascular leakage in different brain sections in experimental model studies. EB dye fluoresces with excitation peaks at 470 and 540 nm and an emission peak at 680 nm. The fluorescence of EB, coupled with the sensitivity of an optical *in vivo* two-dimensional planar fluorescence imaging system (Maestro EX, Caliper Life Sciences, Inc., Hopkinton, MA), that works on the principle of exciting fluorescent dye and recording the intensity of the emitted fluorescence over a range of wavelengths, allowed us to determine the extent of dye accumulation in various brain locations in response to BBB disruption. We used a rat thromboembolic stroke model to validate the usefulness of our optical imaging method for vascular leakage.

## Results

### Plots of EB dye by optical imaging vs. ultraviolet absorbance

**Figure 1:**
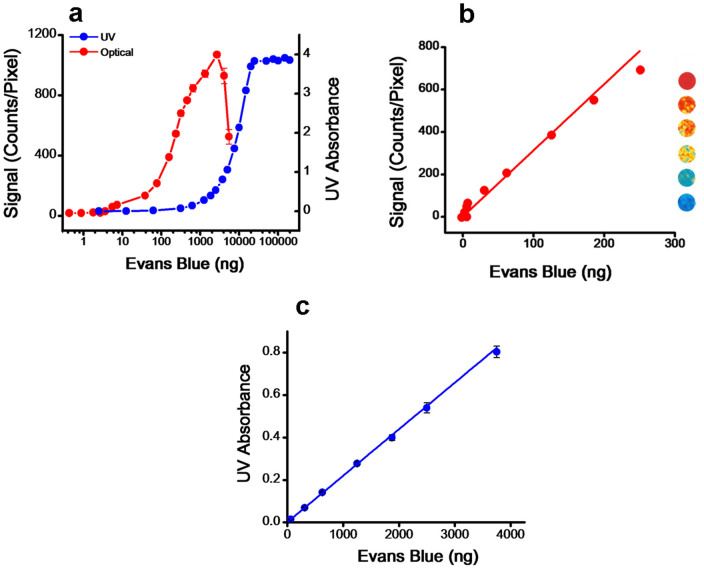
Plots for Evans Blue dye. (a) Changes in optical signal and UV absorbance with EB amount. For optical signal, different amounts of EB dye were loaded onto filter discs. (b) Standard plot for EB dye by optical imaging which is the linear range of the plot “**a**”. Discs exhibited different colors: red indicated the disc loaded with the highest amount of EB dye, blue the disc loaded with the lowest amount. (c) Standard curve for EB dye by UV absorbance, which is the linear range of the plot “**a**”. Data are shown as mean ± s.e.m., *n* = 4.

Recordings of ultraviolet (UV) absorbance and optical signal intensity followed typical sigmoidal curves with increased amounts of EB dye; however, both the methods demonstrated different levels of sensitivity of detection, linear range, and plateau or decline with further increase in EB (Fig. 1a). From these data, we determined that the lowest amount of EB detectable was ~0.05 ng by optical imaging vs. ~62 ng by UV spectrophotometry. Therefore, optical imaging provided >1000-fold higher sensitivity of EB detection than UV spectrophotometry. The plot for optical signal intensity was linear in the range of 0.05 to 355 ng EB dye amount (R^2^ = 0.97; Fig. 1b). Although very sensitive and linear in a wide range (supplementary data for lower and upper range of EB), optical imaging method, like any other analytical methods, may require to use an appropriate range of EB amount for a standard plot to obtain accurate quantitative data in unknown samples. The filter paper discs used for making the EB plots by optical imaging exhibited different colors (heat map) depending on the EB dye amount (Fig. 1b). The plot for EB dye by UV absorbance (Fig. 1c) was linear in the range of 62 ng to 3750 ng EB dye (R^2^ = 0.99). Thus, the linear range for EB dye by UV absorbance was significantly greater than by optical imaging (Fig. 1b vs. 1c). Beyond the linear range, the optical signal showed a decline with further increase in EB, whereas UV absorbance plateaued when it reached the upper limit of the instrument's detection limit (Fig. 1a).

### Mapping of EB dye leakage in brain after induced stroke

Vascular leakage due to BBB breakdown occurs following ischemia-reperfusion injury after stroke. Hence, we validated the effectiveness of optical imaging to map vascular leakage in this model. The neurological score of animals at 3 hrs following induction of stroke was 6 on a scale of 1 to 14 (1–4, mild; 5–9, moderate; and 10–14, severe), indicating that the animals had developed moderate stroke. This is a standard method of scoring stroke animals to determine the extent of brain damage and is quite reproducible. The average infarct volume of the right lobe of the brain, the side that underwent embolic obstruction, determined using TTC staining of brain slices, was 23 ± 2% (mean ± s.e.m., *n* = 10).

**Figure 2:**
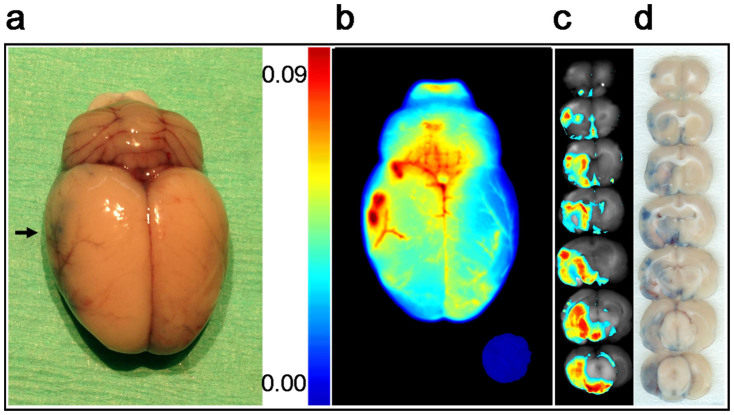
Vascular leakage in stroke. (a) Photographic image of a stroke brain harvested 2 hrs following EB injection which is 5 hr after induction of stroke. Arrow indicates the infarcted side of the brain. (b) Optical imaging of whole brain, areas of leakage is evident from color codes. (c) Optical imaging of brain slices that show vascular leakage. Areas with more leakage show as red, those with less leakage as blue. A filter paper disc loaded with 20 ng EB dye was used as an internal standard. (d) Photographic images of brain slices showing blue color of EB.

The photograph of the entire brain harvested 2 hrs following infusion of EB (5 hrs after stroke induction) showed slightly blue discoloration of the stroke side of the brain due to extravasation of the injected EB dye (Fig. 2a). However, optical imaging of the same brain clearly showed areas of vascular leakage, obtained from the emission of optical signals from the EB accumulated in the infarcted brain (Fig. 2b). Since animals were perfused extensively with saline prior to harvesting the brain, the imaging signal seen is due to the EB localized in the brain tissue. Similar to color codes seen in the standard plot by optical imaging with EB amount (Fig. 1b), the areas with more EB leakage appeared red, those with less leakage as blue-grey, and areas of intermediate leakage appeared yellow (Fig. 2b). The corresponding brain slices showed similar color-coding as in intact brain (Fig. 2c). The regular photographic images of the same brain slices showed blue color of EB but it is difficult to distinguish areas of low or high EB leakage from these images as from optical images (Fig. 2c vs. 2d).

**Figure 3:**
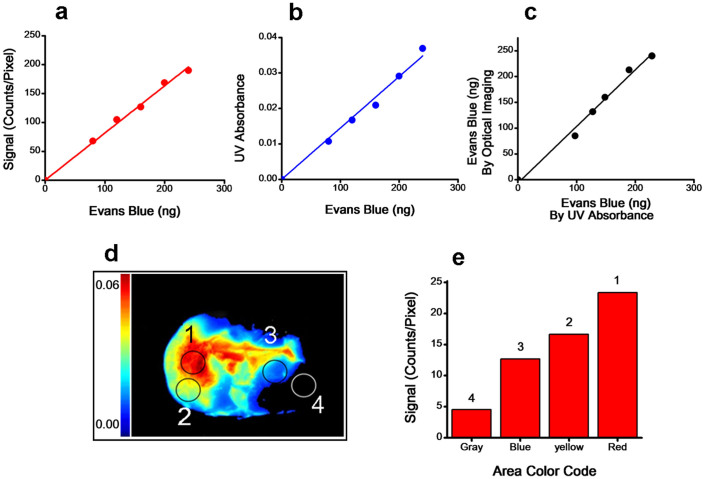
Quantification of optical signals and EB dye in brain slices from a stroke animal. (a) Standard plot for EB amount in brain tissue homogenate using optical imaging. Total signal was normalized to total pixel area for each disk to obtain signal intensity per pixel which is directly correlated to EB amount of the respective disc. (b) Standard plot for EB amount in brain tissue homogenate using UV absorbance. (c) Correlation between EB amount determined by optical and UV absorbance. (d) Optical image of a brain slice from a stroke animal. Circles marked 1–4 of the above brain slices show different color codes depending upon vascular leakage. (e) Quantification of optical signal intensity corresponding to different color regions.

To explore optical imaging to map vascular leakage and quantify EB, we first constructed standard plots of EB in brain tissue by optical imaging (Fig. 3a) and UV absorbance (Fig. 3b). The amounts of EB used for standard plots are in the range conventionally found in the brain following vascular leakage. From the slope of the standard plot, factor to calculate EB amount from optical signal intensity is 111 ng EB per 100 Counts/Pixel. It is important to note here that our standard plots are set in such a way that optical signal intensity (counts/pixel) is directly correlated to EB amount. For the same amount of EB, UV absorbance is ~0.01 which is at the lower end of sensitivity of detection of the spectrophotometer. The EB amounts determined from optical signal intensity and that from UV absorbance show a linear correlation (R^2^ = 0.98) with slope ~1 (slope = 1.05) (Fig. 3c). This correlation confirms that one can quantify EB amount from optical signal intensity. We further validated this by analyzing EB amount in a brain with stroke by both the methods as described below.

To demonstrate the ability of optical imaging to map vascular leakage, we analyzed different areas of a single brain slice (Fig. 3d) for image signal intensity corresponding to different color regions (Fig. 3e). The quantification of EB amount from the optical standard plot (Fig 3a) demonstrated different color codes in the brain slice matching different amount of EB leakage. For example, the brain slice representing area 4 (Gray) corresponded to 4.6 ng EB amount whereas area 1 (Red) corresponded to 23.3 ng EB amount. For the entire brain slice, EB amount was 63.5 ng. This was calculated first by averaging the signal for the entire brain slice to per pixel. The standard plot for optical imaging (Fig. 3a) was used to determine equivalent amount of EB. In a separate experiment, we analyzed EB dye in the entire brain by both optical and UV absorbance methods. Following optical imaging, total EB amount in all the brain slices was determined by UV absorbance. To calculate total EB amount using optical imaging, cumulative signal from all the brain slices was normalized to per pixel area. Using the standard plot for optical imaging, EB amount in the entire brain was 173 ng whereas that determined using UV absorbance was 164 ng. The above data validate the quantitative nature of optical imaging. It is thus feasible to determine EB amount from optical signal intensity in a brain slice, a region of interest or in the entire brain.

### Effect of tissue plasminogen activator on vascular leakage in brain

Currently, administration of tissue plasminogen activator (t-PA), an enzyme that dissolves clots to restore blood flow to ischemic tissue, is the only treatment approved by the US Food and Drug Administration, but t-PA is also known to exacerbate BBB leakage^18^. We tested the effect of t-PA on vascular leakage in normal animals, then in animals that had undergone induced stroke so as to understand the extent of damage that t-PA causes under stroke conditions. In recent studies, it has been shown that t-PA administration via the intracarotid artery is more effective in restoring blood flow to ischemic tissue and hastening recovery than IV injection^19^,^20^. Hence, we tested both routes of administration for t-PA as well as its effect on vascular leakage.

**Figure 4:**
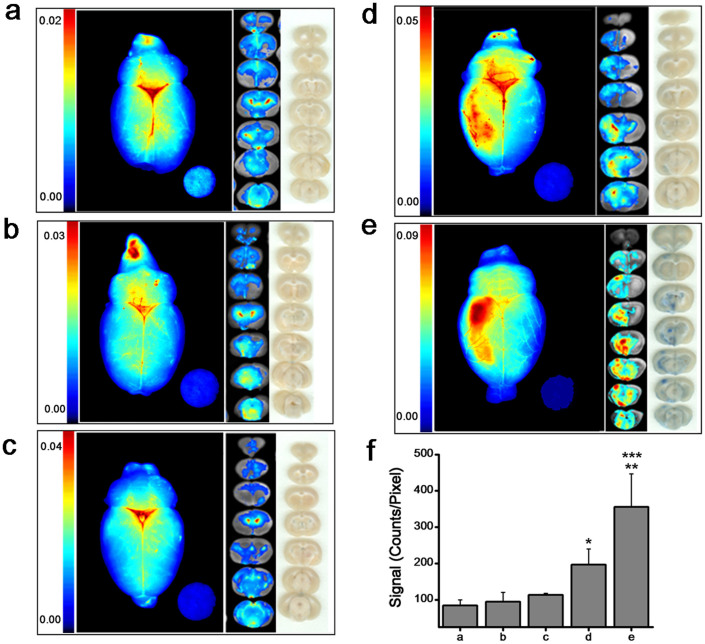
Effect of t-PA on vascular leakage. Normal animals received t-PA either by IV injection via tail vein or intraarterially through the carotid artery. (a) Control animals underwent no surgical procedure except that required for tail vein injection of EB dye. (b) Sham control animals underwent a similar surgical procedure as that used for stroke induction, but saline was injected instead of clot. (c) Animals with t-PA (2 mg/kg) administered via tail vein. (d) Animals with t-PA (2 mg/kg) administered via intracarotid injection. (e) Animals with t-PA (4 mg/kg) administered via intracarotid injection; (f) Quantification of total signal intensities of brain slices from above experiments (a to e). Cumulative signal from all the brain slices was normalized to pixel area. A filter paper disc loaded with 20 ng EB dye was used as an internal standard. Data are shown as mean ± s.e.m., *n* = 4. *p = 0.03, control vs. intracarotid injection of 2 mg/kg t-PA; **p = 0.0001, control vs. intracarotid injection of 4 mg/kg t-PA; ***p, intracarotid injection of 2 mg/kg t-PA vs. 4 mg/kg t-PA. No statistically significant difference between control and sham control (*p* = 0.82) or control and animal injected with 2 mg/kg t-PA via tail vein (*p* = 0.54).

In the control experiment, EB dye was injected without inducing stroke or administering t-PA, and animals did not undergo any surgery except that required for the tail vein injection. These controls showed no significant signal from the EB dye except in areas of the brain where there is no BBB, such as the choroid plexus and circumventricular organs, as seen in brain slices^21^. The whole-brain image showed a trace of EB dye only along the superior sagittal and transverse sinuses; the rest of the brain showed only insignificant signals (Fig. 4a). The data from this experiment served as a control for future comparisons and to test BBB integrity under different experimental conditions. The group of animals that underwent a sham surgery for inducing stroke (only saline injected through the carotid artery instead of a clot) showed no significant differences compared with the above controls (Fig. 4b). Whole brains and brain slices from animals that had received t-PA (2 mg/kg) through tail vein showed no significant EB dye leakage when scanned (HP Scan Jet 3970, Hewlett Packard, Palo Alto, CA). However, optical imaging of the same slices showed a slight increase in signal compared with brain slices from animals that had not received t-PA, but the increase was not statistically significant vs. control (*p* = 0.072; Fig. 4c). Interestingly, when the same dose of t-PA was injected through the carotid artery, scanned images of brain slices revealed EB coloration, which was greater in the right hemisphere than in the left (Fig. 4d). Whole brain and brain slices from animals that had received a double dose of t-PA (4 mg/kg) via the intracarotid route demonstrated intense red coloration on the right side of the brain, the side that had received t-PA (Fig. 4e). Optical images of the whole brain and slices showed lateralization of EB dye leakage, with mosaic colored areas ranging from red to blue in animals which received t-PA via carotid artery (Fig. 4d, e). Quantification of the total signals from all brain slices showed that t-PA administration causes further vascular leakage and the effect depends on the route of administration and dose (Fig. 4f). Using the optical standard plot for EB, we can calculate the amount EB leakage in the entire brain from the cumulative signal intensity from all the brain slices as discussed above. For example, EB brain leakage in animal which received 2 mg/kg t-PA via carotid artery (Fig. 4e) was 62.6 ng whereas this amount was 113.7 ng in the animals which received 4 mg/kg t-PA (Fig. 4e). The fact that brains from animals that had received t-PA showed greater signal intensity than those that had not indicates that EB leakage is due solely to the effect of t-PA, not the surgical procedure.

**Figure 5:**
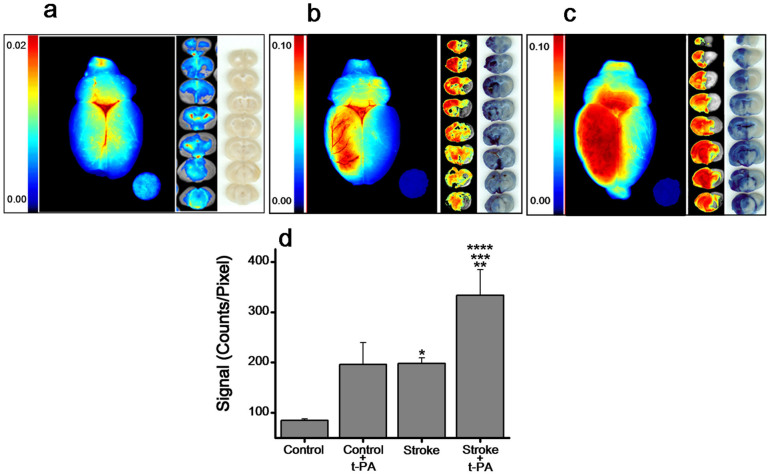
Effect of t-PA on vascular leakage in animals with stroke. (a) Control animals without stroke but received EB. (b) Animals with stroke, imaged 5 hrs after induction of stroke and 2 hrs after EB injection. (c) Animals with stroke that received t-PA (2 mg/kg) administered via intracarotid injection. (d) Quantification of optical signal of all the brain slices from the above groups and normal animal (without stroke, data from Fig. 4d) which received the same dose of t-PA via carotid artery. Data shown are the cumulative signal from all the brain slices normalized to pixel area. A filter paper disc loaded with 20 ng EB dye as an internal standard. Data are shown as mean ± s.e.m., *n* = 3. *p = 0.036, control vs. stroke; **p = 0.001, control vs. stroke + t-PA (2 mg/kg) administered through intracarotid route; ***p = 0.018, stroke vs. stroke + t-PA (2 mg/kg) administered through intracarotid route; ****p = 0.04 control + t-PA vs. stroke + t-PA (2 mg/kg) administered through intracarotid route.

Animals with stroke demonstrated significantly greater vascular leakage than normal animals, with high intensity seen in the core of the infarcted area (Fig. 5a vs. 5b). Administration of t-PA (2 mg/kg via carotid artery) in stroke condition further increased vascular leakage, as evident from the intense dark red areas (Fig. 5b vs. 5c). Comparing signals from normal animals (without stroke induction) that had received the same dose of t-PA, it is clear that administration of t-PA under stroke conditions further aggravates vascular leakage (Fig. 4d vs. Fig. 5c). Quantitative analysis of signal intensities from all brain slices demonstrates a >2.5-fold increase in signal intensities in animals with stroke and a 4.5-fold increase in stroke animals that had also received t-PA vs. sham control animals. The data also show that vascular leakage in normal animals (without stroke) that received t-PA (2 mg/kg) via carotid artery was almost similar to that in animals with stroke (Fig. 5d).

## Discussion

Apart from the high sensitivity of detection, simplicity, and flexibility of analysis, our optical imaging method can quantitatively map EB leakage in different brain areas. This ability may be critical in understanding which part of the brain is most affected. Although UV absorbance method showed a broader linear range for EB amount than optical imaging method (Fig. 1), the EB amount typically found in the brain due to vascular leakage is within the linear range of optical signal intensity whereas it is at a lower end of detection by UV absorbance (Fig. 3). Based on optical imaging analysis, our results show that intracarotid administration of t-PA causes significant vascular leakage even in normal animals (Fig. 4), which is further aggravated in stroke condition (Fig. 5). Optical imaging thus could be very useful for experimental studies in animal models to evaluate BBB integrity in various disease conditions and to test the efficacy of various therapeutic approaches to protect BBB.

The other useful feature of optical imaging software is its ability to perform comparative analysis of images from different protocols, even if those experiments were carried out separately. Furthermore, optical signal intensity does not change with how one groups different images or whether one measures the signal intensity of one image now and compares it later with signal intensities of other images. Although we have used a filter paper disc with fixed amount of EB as an internal standard, there is no need of such an internal standard to compare signal intensity or images for vascular leakage from one experiment to another. Further, one can directly calculate the amount of EB in particular area of the brain or in a brain slice or the entire brain by averaging the optical signal intensity per pixel and using the standard plot, which is directly related to the EB amount. Optical imaging has gained significant interest recently over histological analysis as it can provide a global picture of changes occurring in an organ as well as changes in specific areas of interest. Bahmani et al.^22^ used near-infrared Cy5.5 dye-labeled annexin A5 for noninvasive visualization of cell death in various areas of the brain in a mouse model of stroke.

Recently, Wunder et al.^23^ reviewed various methods for imaging BBB leakage in animal disease models and discussed major obstacles to each technique. These methods either are not sensitive enough or require the use of expensive instrumentation. For example, leakage of gadolinium diethylenetriamine pentaacetic acid through a defective BBB is measured quantitatively using dynamic magnetic resonance imaging (MRI) scanning. The advantages of MRI are that it is noninvasive and one can take repeated scans, which is very useful in a clinical setting, but for animal studies the use of MRI can be quite expensive^24^. Fluorescein isothiocyanate-tagged albumin^25^ or dye-loaded dextran or polymeric nanoparticles (NPs)^26^ have also been used to monitor BBB permeability. Klohs et al.^27^ used near-infrared labeled serum albumin to improve the sensitivity of detection of vascular leakage in stroke animals; however, this method requires synthesizing dye-conjugated albumin. Other drawbacks of these methods include the high dose of labeled albumin required or the need to prevent opsonization of injected NPs so that they remain in the blood circulation long enough to extravasate in sufficient quantity through the leaky vasculature of the brain. Radiotracers used for clinical imaging of BBB impairment require imaging via single-photon emission computed tomography.

We selected a stroke model for our studies because a major pathophysiological change associated with ischemic stroke is BBB disruption^28^. Apart from its utility in stroke, brain tumors, and traumatic brain injuries, the use of EB dye has not been explored extensively to test BBB integrity. This dearth of studies could be because of the low sensitivity of detection of EB dye using conventional methods, either by visual observation of scanned images or analysis of dye content using UV spectrophotometry. In addition, certain biochemical and physiological changes, such as increased levels of inflammatory cytokines in, for example, multiple sclerosis^29^, or of hypoxia-inducible factor-1α, aquaporin-4, or matrix metalloproteinase-9, in other neurological conditions, could cause BBB disruption and hence vascular leakage^30^. Because of high sensitivity and simplicity, we anticipate that our technique could be widely used in research studies involving BBB.

## Methods

### Materials

Evans Blue dye, 2,3,5 triphenyltetrazolium chloride (TTC Stain), thrombin, bovine serum albumin (Sigma-Aldrich, St. Louis, MO), heparin (Hospira, Lake Forest, IL), trichloroacetic acid (TCA) 50% in distilled water (Fisher Scientific, Fair Lawn, NJ), t-PA (Activase®, known generically as alteplase, Genentech, South San Francisco, CA), and 10% phosphate-buffered formalin solution (Cardinal Health, Dublin, OH).

### Standard plots for EB dye constructed from optical imaging and UV spectrophotometry

A stock solution of EB dye (40 mg/ml in normal saline) was diluted serially with saline to obtain dye solutions of desired concentrations. A 5-μl aliquot of each dye solution was placed on a filter paper disc 7 mm in diameter (Whatman Limited, Kent, UK); filter papers were allowed to dry at room temperature for 1 hr. Discs were imaged using the Maestro EX Optical Imaging System (Caliper Life Sciences, Hopkinton, MA). Its blue filter was set between 500 and 720 nm wavelengths for image acquisition, with 787 ms exposure time. The near-infrared filter was set between wavelengths of 740 nm and 950 nm, with 1183.04 ms exposure time. The blue filter captures any autofluorescence and provides an outline of the sample; the near-infrared filter visualizes EB. Total signal intensities of each disc were measured by drawing regions of interest. To obtain a standard plot using UV spectrophotometry, the stock solution of EB dye was diluted with saline to obtain dye solutions of different concentrations. A 5-μl aliquot of each dye solution was mixed with 145 μl of TCA. Samples were loaded into 96-well plates (Thermo Fisher Scientific, Inc., Waltham, MA), and absorbance was recorded using a plate reader at 610 nm wavelength (SpectraMax M2 Multi-Mode Plate Reader, Molecular Devices Corp., Sunnyvale, CA).

### Animal studies

Male Sprague-Dawley rats (400–425 g) were purchased from Harlan Laboratories (Indianapolis, IN). All animal procedures were approved by Cleveland Clinic's Institutional Animal Care and Use Committee, in conformation with federal and institutional guidelines for humane care of animals.

### Validation of optical imaging method for EB amount in brain tissue

Animals were euthanized by an overdose intraperitoneal injection of pentobarbital (150 mg/kg). A midline incision was made, extending from the chin to the lower part of the chest, thoracotomy performed, and the aorta and neck vessels dissected. To flush out blood from the brain, two separate catheters were inserted, one through the aorta to the right common carotid artery (CCA) and the second through the left CCA. These were secured by tie sutures and connected at the other end to an infusion pump (Harvard Apparatus, Holliston, MA) containing heparinized saline (10 U/ml). The infusion was allowed to flow through the body at a rate of 7 ml/min until clear fluid came through the neck vein (~40 ml wash fluid).

Freshly harvested brain was homogenized using Tissue Tearor (Biospec Product, Inc. Bartlesville, OK). To each 100 μl of homogenized brain tissue, known amount of EB dye (stock solution 40 mg/ml) was added and mixed carefully, the samples were incubated at room temperature for 30 min, placed on Maestro Imaging tray, and then imaged as described above. In a second set of experiment, each brain tissue homogenate with a known amount of the EB was mixed with 100 μl TCA (50% TCA mixed in distilled water), centrifuged at 14,000 rpm (Eppendorf Centrifuge 5417R, Eppendorf AG, Hamburg, Germany) for 25 min at 4°C. The supernatant were loaded into 96-well plates (Thermo Fisher Scientific Inc. Waltham, MA), and the absorbance was recorded using a plate reader at 610 nm wavelength (Multimode Plate Reader, Spectra Max, M2, Molecular Device Corp, IA). The data were used to create standard plots; EB amount vs. optical signal and EB amount vs. UV absorbance. These plots were used to calculate the EB amount in stroked brain samples.

### Stroke induction

Stroke was induced using the protocol of Zhenggang et al.^31^ with minor modifications. Anesthesia was induced using 3–4% isoflurane in an induction chamber and maintained using 2.0–2.5% isoflurane via facemask. Under an operating microscope (Leica MZ6, Diagnostic Instruments, Sterling Heights, MI), a ventral midline neck incision was made to expose the right common CCA, external (ECA), and internal carotid arteries (ICA). These arteries were carefully separated from the adjacent vagus nerve. The ECA was ligated with a 6/0 black braided silk suture (Angiotech, Reading, PA) at the distal end and temporarily occluded with a curved microvascular clip (Codman & Shurtleff, Inc., Raynham, MA) at the proximal end. A small incision, very close to the distal end, was made in the ECA wall. A PE-50 modified catheter (internal diameter 0.58 mm, outer diameter 0.96 mm; Becton Dickinson, Sparks, MD) – the tip modified by heating and traction to reduce its internal diameter to 0.30 mm and outer diameter to 0.65 mm – was loaded with 50 U thrombin in 25 μl bovine serum albumin solution (0.1% w/v in saline). The thrombin-loaded catheter was inserted through the ECA and secured with a 6/0 silk suture. The catheter was then advanced slowly to the ICA at a distance of about 17–18 mm until resistance was felt. The catheter was secured by another suture at that point. Arterial blood (10 μl) was drawn slowly into the catheter and allowed to interact with thrombin within the catheter for 20 min to form a clot. The amount of thrombin, blood volume, and time of incubation for clot formation were optimized *ex vivo* to ensure that the clot that formed was of the consistency most likely to cause an embolism. The right and left carotid arties were temporarily occluded with a curved microvascular clip, and the clot was injected slowly over 1 min. The left carotid artery was released after 10 min of clot injection and the right carotid artery after 15 min of clot injection. The wound was closed using a 3/0 silk suture, and the animals were allowed to recover.

### Neurological scoring to assess stroke and determine infarct volume

Neurological assessment and scoring were performed at 3 hrs after clot injection but before injection of EB dye. Animals were evaluated to test their ability to walk in a straight line and to bear weight on the paretic side, as well as to note any circling movements, shaking, or seizures. Motor activities, such as flexion response of the fore- and hindlimbs, as well as the ability of the rat to hold its head within a vertical axis while being raised by the tail, were also evaluated. We assessed sensory responses, such as visual and tactile responses, deep sensory response (response to toe pinch) and other reflex responses, e.g., eye blink in response to touching the cornea with a cotton applicator, pinna reflex by touching the external auditory meatus, and sensitivity to a brief and sudden loud noise by knocking the rat cage^32^. To assess the extent of brain injury, we assigned one point for inability to correctly perform any single motor task or if animals exhibited abnormal behavior such as circling movements, shaking and seizures or showed lack of any reflex response. To score stroke severity, a 14-point checklist was prepared (1–4, mild; 5–9, moderate; and 10–14, severe). This is a scoring method commonly used to assess the extent of stroke in animal models.

Later, to determine infarct volume, brains were excised and brain slices were incubated in a 2% solution of TTC stain dissolved in phosphate-buffered saline (pH 7.4), then incubated in a water bath maintained at 37°C for 15–20 min, transferred to a 10% phosphate-buffered formalin solution, then scanned at 600 dpi resolution using a flatbed scanner (HP ScanJet 3970; Hewlett Packard, Palo Alto, CA). Areas of the infarct lesion were measured using Image J Analysis Software (http://rsb.info.nih.gov/ij/). We manually outlined the margins of the infarcted areas. Unstained areas of brain sections were defined as infarcted if they appeared blue from EB dye leakage. Hemispheric infarcted areas were calculated separately on each coronal slice from 1 to 7, then multiplied by 2 (2-mm thickness of each slice). The summation of these volumes from 1 to 7 represents the hemispheric stroke volume.

### Intraarterial and intravenous administration of t-PA

In animals in which stroke was to be induced, the catheter used for injecting the clot was retained in place to be used later to inject t-PA. Control animals (no induced stroke) underwent surgery for right ICA catheterization as described above for induction of stroke. A PE 10 catheter (internal diameter 0.28 mm, outer diameter 0.61 mm; Becton Dickinson, Sparks, MD) was inserted through the ICA for t-PA administration. A bolus of 10% of the total dose of t-PA (2 mg/kg or 4 mg/kg; t-PA solution = 1 mg/ml) was given, then the remaining dose was infused slowly over 40 min using an infusion pump (Harvard Apparatus, Holliston, MA). In animals with stroke, t-PA was administered as noted above 3 hrs after induction of stroke, followed by EB dye injection as described below. For IV injection, 2 mg/kg t-PA was injected through the tail vein as a bolus. For t-PA injection, the tail was cannulated under an infrared heating lamp while the animal was placed on a heating pad under anesthesia induced using 2.0–2.5% isoflurane via facemask. A rubber band was tied around the tail to make the vein engorged. The area to be punctured was cleaned with an alcohol pad. A 26-gauge cannula (Hospira, Lake Forest, IL) was inserted through the tail vein and secured with adhesive tape. This point of the IV access was also used for EB dye injection (2.5 ml/kg, 4% w/v in saline) in all experimental animals. Animals were euthanized 2 hrs following this dye injection with an overdose injection of pentobarbital (150 mg/kg) by the intraperitoneal route. The control experiment involved injecting dye solution in animals that had undergone the sham intraarterial injection procedure but received saline instead of t-PA through the carotid artery. In another set of controls, dye was injected without performing surgery to determine the effect of surgery itself on BBB integrity. To clear the dye, vessels of the euthanized animals were flushed with heparinized saline as described above.

### Evans blue dye leakage

We took digital photographs (Canon Power Shot S5IS, Japan) of the harvested brains, then immediately took optical images using the Maestro system under the same setting as described above for standard plots. Brains were sectioned coronally into seven 2-mm-thick slices, from rostral to caudal (Brain Matrix, Electron Microscopy Sciences, Hatfield, PA). The sections were imaged using Maestro under the same setting as above, and the signal intensity was recorded. A filter paper disc loaded with 20 ng amount of EB dye as an internal standard. Following optical imaging, the EB dye content of the brain was quantified by UV spectrophotometry. Brain slices were homogenized, processed as above for EB amount.

### Statistical analysis

All numerical data are expressed as mean ± standard error of the mean. Statistical significance was determined by one-way ANOVA with multiple comparisons. Statistical significance was set at *p* < 0.05.

## Author Contributions

H.J. carried out animal studies, I.M.A. optimized imaging protocol, and V.L. initiated the idea and supervised the study. All authors contributed to writing the manuscript and reviewed the manuscript prior to submission.

## Supplementary Material

Supplementary Information Supplementary Information

## References

[b1] Rubin L. L. & Staddon J. M. The cell biology of the blood-brain barrier. Annu. Rev. Neurosci. 22, 11–28 (1999). 10202530 10.1146/annurev.neuro.22.1.11

[b2] Kroll R. A. & Neuwelt E. A. Outwitting the blood-brain barrier for therapeutic purposes: osmotic opening and other means. Neurosurgery 42, 1083–1099; discussion 1099–1100 (1998). 9588554 10.1097/00006123-199805000-00082

[b3] Manda V. K., Mittapalli R. K., Bohn K. A., Adkins C. E. & Lockman P. R. Nicotine and cotinine increases the brain penetration of saquinavir in rat. J. Neurochem. 115, 1495–1507 (2010). 20950334 10.1111/j.1471-4159.2010.07054.x

[b4] Blanchette M. & Fortin D. Blood-brain barrier disruption in the treatment of brain tumors. Methods Mol. Biol. 686, 447–463 (2011). 21082387 10.1007/978-1-60761-938-3_23

[b5] Qin Y. *et al.* Comparison of four different peptides to enhance accumulation of liposomes into the brain. J. Drug Target. 20, 235–245 (2012). 22188312 10.3109/1061186X.2011.639022

[b6] Konofagou E. E. *et al.* Ultrasound-induced blood-brain barrier opening. Curr. Pharm. Biotechnol. 13, 1332–1345 (2012). 22201586 10.2174/138920112800624364 PMC4038976

[b7] Johansen C. K., Welker K. M., Lindell E. P. & Petty G. W. Cerebral corticospinal tract injury resulting from high-voltage electrical shock. Am. J. Neuroradiol. 29, 1142–1143 (2008). 18372420 10.3174/ajnr.A1009 PMC8118827

[b8] Risling M. & Davidsson J. Experimental animal models for studies on the mechanisms of blast-induced neurotrauma. Front. Neurol. 3, 30 (2012). 22485104 10.3389/fneur.2012.00030 PMC3317041

[b9] Ruda R., Trevisan E. & Soffietti R. Epilepsy and brain tumors. Curr. Opin. Oncol. 22, 611–620 (2010). 20706121 10.1097/CCO.0b013e32833de99d

[b10] McDannold N., Vykhodtseva N. & Hynynen K. Blood-brain barrier disruption induced by focused ultrasound and circulating preformed microbubbles appears to be characterized by the mechanical index. Ultrasound Med. Biol. 34, 834–840 (2008). 18207311 10.1016/j.ultrasmedbio.2007.10.016 PMC2442477

[b11] Ichikawa H. & Itoh K. Blood-arachnoid barrier disruption in experimental rat meningitis detected using gadolinium-enhancement ratio imaging. Brain Res. 1390, 142–149 (2011). 21435335 10.1016/j.brainres.2011.03.035

[b12] Huppert J. *et al.* Cellular mechanisms of IL-17-induced blood-brain barrier disruption. FASEB J. 24, 1023–1034 (2010). 19940258 10.1096/fj.09-141978

[b13] Ladewig G. *et al.* Spatial diversity of blood-brain barrier alteration and macrophage invasion in experimental autoimmune encephalomyelitis: a comparative MRI study. Exp. Neurol. 220, 207–211 (2009). 19733560 10.1016/j.expneurol.2009.08.027

[b14] Kim J. E., Ryu H. J., Choi S. Y. & Kang T. C. Tumor necrosis factor-alpha-mediated threonine 435 phosphorylation of p65 nuclear factor-kappaB subunit in endothelial cells induces vasogenic edema and neutrophil infiltration in the rat piriform cortex following status epilepticus. J. Neuroinflammation. 9, 6 (2012). 22240205 10.1186/1742-2094-9-6 PMC3312845

[b15] Gartshore G., Patterson J. & Macrae I. M. Influence of ischemia and reperfusion on the course of brain tissue swelling and blood-brain barrier permeability in a rodent model of transient focal cerebral ischemia. Exp. Neurol. 147, 353–360 (1997). 9344560 10.1006/exnr.1997.6635

[b16] Latour L. L., Kang D. W., Ezzeddine M. A., Chalela J. A. & Warach S. Early blood-brain barrier disruption in human focal brain ischemia. Ann. Neurol. 56, 468–477 (2004). 15389899 10.1002/ana.20199

[b17] Ballabh P., Braun A. & Nedergaard M. The blood-brain barrier: an overview: structure, regulation, and clinical implications. Neurobiol. Dis. 16, 1–13 (2004). 15207256 10.1016/j.nbd.2003.12.016

[b18] Caplan L. R. Tissue Plasminogen Activator for Acute Ischemic Stroke. New Engl. J. Med. 333, 1581–1588 (1995). 7477192 10.1056/NEJM199512143332401

[b19] Alexandrov A. V. & Grotta J. C. Arterial reocclusion in stroke patients treated with intravenous tissue plasminogen activator. Neurology 59, 862–867 (2002). 12297567 10.1212/wnl.59.6.862

[b20] Qureshi A. I. *et al.* Reocclusion of recanalized arteries during intra-arterial thrombolysis for acute ischemic stroke. Am. J. Neuroradiol. 25, 322–328 (2004). 14970040 PMC7974601

[b21] Gilgun-Sherki Y., Melamed E. & Offen D. Oxidative stress induced-neurodegenerative diseases: the need for antioxidants that penetrate the blood brain barrier. Neuropharmacology 40, 959–975 (2001). 11406187 10.1016/s0028-3908(01)00019-3

[b22] Bahmani P. *et al.* Visualization of cell death in mice with focal cerebral ischemia using fluorescent annexin A5, propidium iodide, and TUNEL staining. J. Cereb. Blood Flow Metab. 31, 1311–1320 (2011). 21245871 10.1038/jcbfm.2010.233 PMC3099638

[b23] Wunder A., Schoknecht K., Stanimirovic D. B., Prager O. & Chassidim Y. Imaging blood-brain barrier dysfunction in animal disease models. Epilepsia 53 Suppl 6, 14–21 (2012). 23134491 10.1111/j.1528-1167.2012.03698.x

[b24] Tofts P. S. & Kermode A. G. Measurement of the blood-brain barrier permeability and leakage space using dynamic MR imaging. 1. Fundamental concepts. Magn. Reson. Med. 17, 357–367 (1991). 2062210 10.1002/mrm.1910170208

[b25] Suidan G. L., McDole J. R., Chen Y., Pirko I. & Johnson A. J. Induction of blood brain barrier tight junction protein alterations by CD8 T cells. PLoS One 3, e3037 (2008). 18725947 10.1371/journal.pone.0003037 PMC2516328

[b26] Provenzale J. M., Mukundan S. & Dewhirst M. The role of blood-brain barrier permeability in brain tumor imaging and therapeutics. Am. J. Roentgenol. 185, 763–767 (2005). 16120931 10.2214/ajr.185.3.01850763

[b27] Klohs J. *et al.* Near-infrared fluorescence imaging with fluorescently labeled albumin: a novel method for non-invasive optical imaging of blood-brain barrier impairment after focal cerebral ischemia in mice. J. Neurosci. Methods 180, 126–132 (2009). 19427539 10.1016/j.jneumeth.2009.03.002

[b28] Liu J., Jin X., Liu K. J. & Liu W. Matrix metalloproteinase-2-mediated occludin degradation and caveolin-1-mediated claudin-5 redistribution contribute to blood–brain barrier damage in early ischemic stroke stage. J. Neurosci. 32, 3044–3057 (2012). 22378877 10.1523/JNEUROSCI.6409-11.2012 PMC3339570

[b29] D'Aversa T. G., Eugenin E. A., Lopez L. & Berman J. W. Myelin basic protein induces inflammatory mediators from primary human endothelial cells and blood-brain barrier disruption: implications for the pathogenesis of multiple sclerosis. Neuropathol. Appl. Neurobiol. 39, 270–283 (2013). 22524708 10.1111/j.1365-2990.2012.01279.x PMC3430818

[b30] Wang Z. *et al.* Potential contribution of hypoxia-inducible factor-1alpha, aquaporin-4, and matrix metalloproteinase-9 to blood-brain barrier disruption and brain edema after experimental subarachnoid hemorrhage. J. Mol. Neurosci. 48, 273–280 (2012). 22528459 10.1007/s12031-012-9769-6

[b31] Zhang Z. *et al.* A new rat model of thrombotic focal cerebral ischemia. J. Cereb. Blood Flow Metab. 17, 123–135 (1997). 9040491 10.1097/00004647-199702000-00001

[b32] Li Y., Chen J., Wang L., Lu M. & Chopp M. Treatment of stroke in rat with intracarotid administration of marrow stromal cells. Neurology 56, 1666–1672 (2001). 11425931 10.1212/wnl.56.12.1666

