# Supplementary material for: Optical imaging to map blood-brain barrier leakage
Source: Sci Rep. 2013 Nov 1;3:3117. doi: 10.1038/srep03117 (PMC3814906; doi:10.1038/srep03117)
Supplement: Supplementary Information [file srep03117-s1.pdf]

Supplementary Information

Optical imaging to map blood-brain barrier leakage

Hayder Jaffer, Isaac M. Adjei, and Vinod Labhasetwar*

Department of Biomedical Engineering, Lerner Research Institute,

Cleveland Clinic, Cleveland, OH 44195

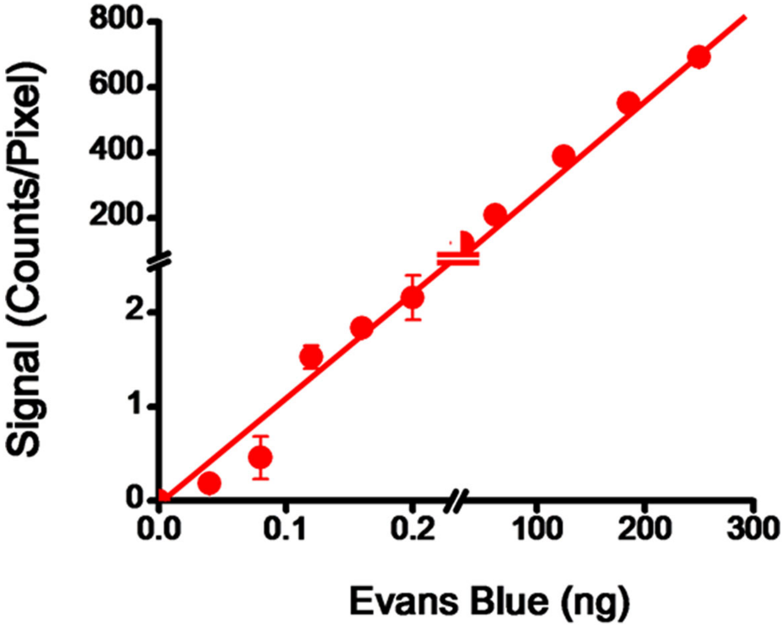

Standard plot for Evans Blue: Plot shows optical signal intensity for Evans Blue (EB) at lower range (0.0 ng to 0.2 ng, R^2 =0.98), higher range (62 ng to 250 ng, R^2 =0.99), and entire range (0.0 ng to 250 ng, R^2 = 0.97). The method used for constructing plots was the same as described in the manuscript. The data show high sensitivity and wide linear range of detection of EB using optical imaging method.
